# Supplementary material for: ‘Wish you were here’: Managers’ experiences of hybrid work in higher education
Source: PLoS One. 2025 Dec 17;20(12):e0339120. doi: 10.1371/journal.pone.0339120 (PMC12711030; doi:10.1371/journal.pone.0339120)
Supplement: Attachment — Submitted filename: Response to editor.docx [file pone.0339120.s003.docx]

PONE-D-25-27056R1

‘Wish you were here’: Managers’ experiences of hybrid work in higher education

PLOS ONE

Dear Editor,

Thank you for your positive response and constructive comments on our resubmission. We have now accordingly revised the manuscript. Our detailed point-by-point responses, addressing the remaining concerns, are provided below.

First, I suggest some clarifications in the abstract. It is true that telework research has generally placed more emphasis on employees—yet managers are also workers, and the current phrasing of that part of the abstract could be somewhat misleading. I would also recommend concisely describing the sample in the abstract: how many participants were interviewed, who these managers are, and the sector or tasks involved. Please be explicit that this is a sample of managers employed in different services at a Swedish medical university.

*- Thank you for providing us with this opportunity*. We have rephrased the mentioned sentence in the abstract to avoid (unintentionally) suggesting that managers are not employees (please see lines 31-32). *Further clarifications on the participants and the setting have also been added to the abstract on lines 32-35.*

At present, the abstract remains unchanged from the original version and could be more informative. Rather than focusing mainly on the analytical categories emerging from the narrative data—which are quite common in similar analyses—it would be ideal to include a concise summary of the main findings. As this is a qualitative study, we expect more nuance, new mechanisms, and a finer-grained understanding of these managers’ experiences.

*- The summary of the conclusions is further developed in the abstract (lines 40-48) to highlight that this study provides a more fine-grained understanding of managers' experiences.*

Regarding the analytical technique, I am not familiar with the term “conventional content analyis.” It seems to me that it would be more appropriate to frame this as a Qualitative Content Analysis (QCA) (see Hsieh HF, Shannon SE. Three approaches to qualitative content analysis. Qual Health Res. 2005;15(9):1277–1288. doi:10.1177/1049732305276687; PMID: 16204405). In any case, it would be useful to add a methodological reference clarifying the data analysis.

*- We have rephrased the abstract (line 38) and the methods section (lines 215-224) to clarify that the method used is a qualitative content analysis (QCA). However, following Hsieh and Shannon (2005, p. 1277), we also think it is important to show which of the three distinct main approaches they list (conventional, directed, and summative) we have used, namely “the conventional content analysis approach”. We have clarified this by consistently using the word 'approach' when introducing and referring to “the conventional content analysis approach,” and by adding the reference again when describing how it has structured the data analysis (lines 215-224).*

Finally, the title is attractive—and although I am a Pink Floyd fan—the message it conveys seems to suggest that managers are unhappy with hybrid work. Your results shows many challenges but appear to be more nuanced. I am ok with keeping the title, but I would therefore recommend justifying it more explicitly in the discussion or adjusting it to better reflect your findings.

*- Thank you for giving us this opportunity to develop our results, further nuance managers' experiences, and keep the title. To further illustrate managers’ wish for on-site presence to facilitate their own leadership roles and tasks, while simultaneously appreciating the benefits their employees enjoy in hybrid work, subsequent changes have been made in the discussion (481-484) and in the abstract (lines 46-48).*
